# A cross-sectional, exploratory survey on health-relevant free-time activities and body mass index in preschool children in urban and rural settings of Austria

**DOI:** 10.1186/s12887-021-02972-x

**Published:** 2021-11-06

**Authors:** J. Robatsch, P. Voitl, Susanne C. Diesner-Treiber

**Affiliations:** 1 First Vienna Pediatric Medical Center, Donau-City Strasse 1, 1220 Vienna, Austria; 2 Sigmund Freud University Vienna, Donau-City Strasse 1, 1220 Vienna, Austria

**Keywords:** Preschoolers, Free-time activities, Obesity, Sedentary behaviors, Physical activity

## Abstract

**Background:**

The increasing prevalence of obesity is among the most relevant healthcare issues in Europe. The number of overweight people rises due to lifestyle changes, increased sitting activities, and less physical activity. Prevention in early childhood is paramount to stop this alarming trend.

**Aim:**

This study primarily aimed to evaluate the average time children (3-5 years) from rural and urban Austrian regions spent engaging in physical activity and sedentary behaviors in their free-time. Additionally, we investigated the potential correlation between duration and habits of free-time activity or place of residence and age- and sex-specific body mass index (BMI). The potential impact of socio-economic factors on BMI was examined.

**Methods:**

Urban (Vienna) and rural (Carinthia) regions of Austria were chosen for this observational cross-sectional study. Preschool children (n=130) attending nurseries in these regions were included. Weight and height were measured and BMI calculated. Free-time activity and socio-economic data were asked using a self-administered questionnaire. Data on sedentary behavior time (sedentary activity and media consumption) and physical activity time (defined as organized or spontaneous exercise) were analyzed using non-parametric tests.

**Results:**

Preschool children spent approximately as many hours of their free-time engaged in physical activity as in sedentary behaviors. Time trend in media consumption amounts to one-third of the cumulative time spent engaging in sedentary behaviors. Preschoolers from the urban area spent fewer hours practicing organized exercise and more in sedentary behaviors than peers in the rural area. In the selected areas, 7 % of preschoolers were overweight, 3.9 % were obese. BMI was not associated with free-time activities but showed a trendwise negative correlation with organized exercise. A positive correlation of age and organized exercise was observed but not with physical activity per se.

**Conclusions:**

Our results confirm the necessity of preventive interventions among Austrian preschoolers and lead to a better understanding of their free-time activities. Further investigations with larger study populations are needed to promote effective childhood obesity prevention and examine the differences regarding obesity prevalence and leisure-time activity between rural and urban areas.

**Supplementary Information:**

The online version contains supplementary material available at 10.1186/s12887-021-02972-x.

## Background

Overweight and obesity are among the major health problems of the 21st century [1, 2]. The WHO classifies the nutritional status based on the body mass index into underweight (BMI <18.5 kg/m2), normal weight (18.5-24.9 kg/m2), overweight (BMI ≥25 kg/m2) and obesity (BMI ≥30 kg/m2) in adults [3]. BMI values ​​for children and adolescents are expressed as sex- and age-specific percentiles, as a standard deviation score (SD-score) or relative BMI (rBMI) [2–4]. Due to the statistical distribution of BMI values ​​in German-speaking countries, the overweight limit ​​for children and adolescents between 0 and 18 years is a BMI >90th percentile (approx. +1 SD-score), the obesity value exceeds the 97th percentile (approx. +2 SD-score) and extreme obesity 99.5th, while the cut-off for underweight is the 10th BMI-percentile [4–6]. Normal weight lies between the 10th and 90th BMI-percentile. At the age of 18, percentiles switch to the corresponding risk-related BMI limit values ​​for adults as a transition to definitions for different age groups [5, 7].

According to the World Health Organization (WHO) in 2016, 18 % of children and adolescents between 5 and 19 worldwide were overweight, 6 % of girls and 8 % of boys were obese [1]. The increasing prevalence of overweight in children and adults around the world continually presents our healthcare systems with new economic, prevention, and therapeutic challenges.

The multifactorial causes of the rising prevalence of obesity in the last decades involve a lifestyle with insufficient physical activity and excessive consumption of high-caloric food. Several socio-economic factors, as well as pre- and perinatal determinants, increase the risk of obesity [8–12].

Obesity is not only a risk factor and contributor to increase morbidity and mortality in adults but affects already the children’s wellbeing [2, 5, 13]. Common obesity-related diseases, such as hypertension, lipid metabolism disorders, type 2 diabetes mellitus, or musculoskeletal system disorders, can affect children and adolescents and are associated with increased morbidity and mortality in the long term [2, 7]. Asthma, obstructive sleep apnea, infertility, polycystic ovary syndrome, and psychiatric disorders are also associated with an elevated BMI [14]. Discrimination and bullying, social isolation, and low self-esteem deriving from the stigma around obesity, along with body consciousness as early as in childhood, contribute significantly to severe psychological issues: anxiety, mood disorders, or disturbed eating behaviors such as binge eating [15–17].

As overweight children are more likely to become overweight adults [18, 19] lifestyle habits, such as insufficient physical activity or dietary patterns, which are shaped at a young age, might be maintained later in life [19]. Therefore, it is necessary to understand that overweight and obesity are mostly preventable but early life preventive measures are needed. Nursery coincides with a critical window- thus the importance of a better understanding of preschoolers’ free-time activities as a potential target of obesity prevention is important, as confirmed by other results showing after-school time to play an essential role in establishing health-promoting activities in everyday life [20, 21].

Data on the correlation between physical activity and weight in children and adolescents is mostly incongruent [22–25]. Not all children and adolescents accumulate the physical activity time recommended by the WHO being (60 min per day for children aged 5-17) [1]. Sedentary behavior and extensive screen media are unhealthy and represent a challenging habit for parents to break. Sedentary behaviors remain undoubtedly associated with obesity in many studies [25], while the effect of media exposure on BMI are discussed controversially in the corresponding literature [26–30].

The prevalence of obesity increases regardless of the age groups and might have some association with the place of residency as according to international data the prevalence of overweight and obesity is differently distributed in rural and urban regions [1, 2, 31–36].

In our opinion healthy early life free-time activities are a prerequisite for a healthy development and BMI not only at that age but also later in life. Thus, in this descriptive cross-sectional study we aimed to evaluate the average time children aged 3-5 years from rural and urban Austrian regions spent engaging in physical activity and sedentary behaviors in their free-time. We then analyzed if the duration of free-time activities was associated with age- and sex-specific BMI. The potential correlation of socio-economic factors, such as education level, the parents’ employment, the residence (urban or rural region) on BMI was examined.

The results might contribute to a better understanding of preschoolers’ free-time activities in these examined regions and can be the basis for further studies to provide indispensable insights into obesity prevention and healthy life style habits early in life.

## Methods

### Study design and setting

The current study is a cross-sectional exploratory survey, aiming to primarily compare free-time activities and collect data on BMI and socio-economic factors of preschool children in chosen rural and urban areas of Austria. Due to the exploratory design, sample size calculation was not performed.

This study considers cities with more than 100.000 inhabitants as urban areas and communities with 5.000 inhabitants or less as rural areas. In Austria big cities are defined as >100.000 inhabitants while a clear definition for rural areas does not exist. We defined rural areas as described by the Food and Agriculture Organization of the United Nations, indicating that 5.000 or less inhabitants come form rural clusters [37, 38].

We have chosen 4 nurseries located in different districts of Vienna, representing urban regions, and 6 nurseries in several communities in Carinthia, representing rural areas.

### Study population

The study population includes apparently healthy children aged 3 to 5 years, attending one of the addressed nurseries, as well as their parents or legal guardians. Infants born prematurely and children with known chronic diseases were excluded.

### Questionnaire

After consenting to participate, the parents or legal guardians filled in a self-administered questionnaire at the respective nurseries to provide demographic and socio-economic data of their preschool children (Supplementary data questionnaire). The study team collected data during visits to the preschools where a trained study member measured weight (kg) and height (cm) of the children in light clothes, without shoes [39], upright position and with the same instruments (digital scale brand AICOK, model no. YHF1431; measuring tape fixated on portable stadiometer). The weight and height were measured with an accuracy of one decimal number.

The Ethics Committee of the Medical University of Vienna approved this study (EK Nr. 1544/2017). Participation was voluntary and anonymous. Written informed consent to participate in this study was provided by the participants’ legal guardian / next of kin.

### Measures

Although the questionnaire was developed by the authors themselves the choice of questions were based on validated study designs [40–43]. Parents reported the following parameters (1+2) and estimated their preschool children’s time performing different free-time activities. The BMI (3) was calculated from the measured height and weight values. The detailed questions are listed in the supplementary file (Supplementary data questionnaire).

- How many hours per week were devoted on average to the following free-time activities: Physical activity defined as organized exercise (such as sport programs with consistent attendance, i.e., dance class, or gymnastics, hours per week) and spontaneous exercise (such as additional physical exertion like running, biking, playground activities, hours per week). Sedentary behaviors defined as sedentary activity (such as playing with toys, drawing, hours per week) and screen media consumption (i.e., “screen-time”: TV, computer, tablet, smartphone, hours per week) (Supplementary Fig. 1);
- Socio-economic factors: region (rural/urban; i.e. Carinthia/Vienna), age (years), gender (male, female), birth order (firstborn; middle child; lastborn), age of parents (years), parental occupational status (yes/no), highest parental education (less than high school completion; high school diploma; university degree).
- Body-mass-index (calculated from measured height (cm) and weight (kg) values): raw values and weight classes (underweight BMI ≤10th percentile; normal weight 10th < BMI <90th percentile; overweight without obesity 90th ≤ BMI < 97th percentile; obesity BMI ≥97th percentile) grouped according to percentiles after Kromeyer-Hauschild et al. 2001 [4]..

### Statistical analysis

Data analysis was performed using Microsoft Excel and IBM SPSS® software. Kolmogorov-Smirnov-Test and Shapiro-Wilk-Test were used to test normal distribution, absent in most of the tested variables. Analysis of categorial variables included frequencies in absolute numbers and percentages for the total study population and regions separately and for all categories of ordinal variables, as well as Fisher’s Exact Test. Descriptive analysis of quantitative variables included median values, minimum and maximum for the total study population and regions separately. Two-sided *p*-values of Wilcoxon-Mann-Whitney-U-Test were calculated to test the differences of free-time activities between the rural and urban regions. Wilcoxon signed-rank test was performed to assess the differences between physical activity and sedentary behavior within a region. Kruskal-Wallis-Test was used to determine the variations concerning activities among weight classes. Correlations between age and free-time activities were calculated using the non-parametric coefficient of Spearman-*Rho*.

Non-parametric data were transformed using the Johnson transformation a method for achieving normal distribution [44]. These data were then used for linear regression analysis with BMI as the dependent variable. Normal distribution of residuals was confirmed. Regression analysis was conducted to test the correlation of socio-economic factors and BMI. Strength, direction and significance of the effects are shown as regression coefficient *B* (95 % CI), Beta and *p*-value (Supplementary Table 1). Bonferroni correction was performed for multiple comparisons. *P*-values <0.05 were considered statistically significant.

The study was conducted according to the STROBE-Statement on observational studies for evidence-based medicine [45].

## Results

### Cohort characterization

This cross-sectional observational study involved four nurseries in four different municipal districts of Vienna (urban area) and six nurseries in six municipalities of Carinthia (rural area). This pilot study is conceived as a guide for future large-scale analyses to address questions that were not thoroughly answered here. Opening hours had to be similar (6:30 or 7:00 a.m. - 4:00 p.m. and 5:30 p.m., Vienna, on average 10.88 h / day vs. Carinthia 9.66 h / Open every day), to ensure comparability of marginal conditions.

Out of 130 children included in the study, 58 were from Vienna, 72 were from Carinthia, 65 were female, and 65 were male. No significant sex differences between participants from rural and urban nurseries were observed.

The characteristics of the study cohorts can be found in Tables 1 and 2.

**Table 1:** Descriptive data analysis I: Child

		Total	Urban	Rural	***p***	***p***-adjust
		(*n* = 130)	(*n* = 58)	(*n* = 72)		
**Gender**	male	50% (65)	51.7% (30)	48.6% (35)	0.7284	1
	female	50% (65)	48.3% (28)	51.4% (37)		
**Age**	years	4.5 (3-5.5)	4 (3-5.5)	4.5 (3-5.5)	0.059	0.649
**Birth order**	firstborn	48.5% (63)	50% (39)	47.2% (34)	0.3288	1
	lastborn	41.5% (54)	43.1% (25)	40.3% (29)		
	other	10% (13)	6.9% (4)	12.5% (9)		
**Weight**	kilogramm	18 (11.9-30.3)	16.5 (12.9-25.8)	18.7 (11.9-30.3)	0.149	1
**Height**	meters	1.07 (0.9-1.23)	1.05 (0.9-1.22)	1.1 (0.92-1.23)	0.435	1
**BMI**	kg/m2	15.82 (12.42-20.83)	15.83 (12.42-20.83)	15.81 (13.06-20.34)	0.535	1
**Weight class**	underweight	4.7% (6)	7% (4)	2.8% (2)	0.4558	1
	normalweight	84.5% (109)	86% (49)	83.3% (60)		
	overweight w/o obesity	7% (9)	5.3% (3)	8.3% (6)		
	obesity	3.9% (5)	1.8% (1)	5.6% (4)		
**Physical activity**	hours/week	12 (0-40)	12 (4-40)	12 (0-32)	0.746	1
Organized exercise	hours/week	0 (0-10)	0 (0-5)	1 (0-10)	0.001*	0.016*
Spontaneous exercise	hours/week	10 (2-40)	11 (3-40)	10 (2-30)	0.381	1
**Sedentary behaviors**	hours/week	13 (1-34)	14.5 (4-34)	10.75 (1-30)	0.010*	**0.13**
Sedentary activity	hours/week	7 (0-28)	10 (0-21)	7 (1-28)	0.030*	**0.36**
Media consumption	hours/week	4 (0-16)	5 (0-16)	4 (0-10)	0.007*	**0.098**

Categorial data: frequency in%, (absolute number); 2-sided *p*-value Fisher’s Exact Test; Quantitative data: median (min - max); 2-sided *p*-value Wilcoxon-Mann-Whitney U test; * *p*-value <0.05. *p*-adjust value after Bonferroni correction. Urban n = 58, rural n = 72, except for Physical activity: rural n = 81

**Table 2:** Descriptive data analysis II: Parents

		Total	Urban	Rural	***p***	***p***-adjust
		(*n* = 130)	(*n* = 58)	(*n* = 72)		
**Age mother**	years	34 (22-51)	34 (22-47)	34 (25-51)	0.576	1
**Age father**	years	36 (24-62)	36 (24-55)	36 (24-62)	0.596	1
**Employment mother**	no	18% (23)	15.8% (9)	19.7% (14)	0.647	1
	yes	82% (105)	84.2% (48)	80.3% (57)		
**Employment father**	no	5.6% (7)	7.1% (4)	4.4% (3)	0.7	1
	yes	94.4% (117)	92.9% (52)	95.6% (65)		
**Highest education level mother**	< high school diploma	40.6% (52)	32.8% (19)	47.1% (33)	0.249	1
	high school diploma	31.3% (40)	36.2% (21)	27.1% (19)		
	university	28.1% (36)	31.0% (18)	25.7% (18)		
**Highest education level father**	< high school diploma	43.5% (54)	25.9% (15)	59.1% (39)	0.001*	0.006*
	high-school diploma	26.6% (33)	32.8% (19)	21.2% (14)		
	university	29.8% (37)	41.4% (24)	19.7% (13)		

Categorial data: frequency in % (absolute number); 2-sided *p*-value Fisher’s Exact Test; Quantitative data: median (min – max); 2-sided *p*-value Wilcoxon-Mann-Whitney U test; * *p*-value <0.05, *p*-adjust value after Bonferroni correction

### Prevalence of overweight and obesity in selected Austrian cohorts

In the Austrian regions selected for this study, 84.5 % of preschoolers are a healthy weight (between 10th and 90th percentiles), 4.7 % are underweight (definition BMI <= 10th percentile), 7 % overweight not obese (90th ≤ ​​BMI < 97th percentile) and 3.9 % of the preschoolers fall within the obesity range (BMI ≥97th percentile). No significant differences were observed between the considered rural and urban regions regarding overweight and obesity (*p*=0.456, see Table 1).

### Time spent on physical activity and sedentary behaviors

The amount of free-time preschoolers spend performing physical activity is statistically comparable to the time dedicated to sedentary behavior (Wilcoxon sign test, *p*=0.778).

As it turns out, spontaneous exercise (such as additional physical exertion like running, biking, playground activities) is more customary among preschoolers than organized exercise (such as club sports, soccer, gymnastics, dance lessons). According to their parents, preschoolers devote about twice as many hours of their weekly free-time to sedentary activities (such as drawing, playing with toys) than to media consumption.

### Free-time activities in urban and rural regions of Austria

**Fig. 1:**
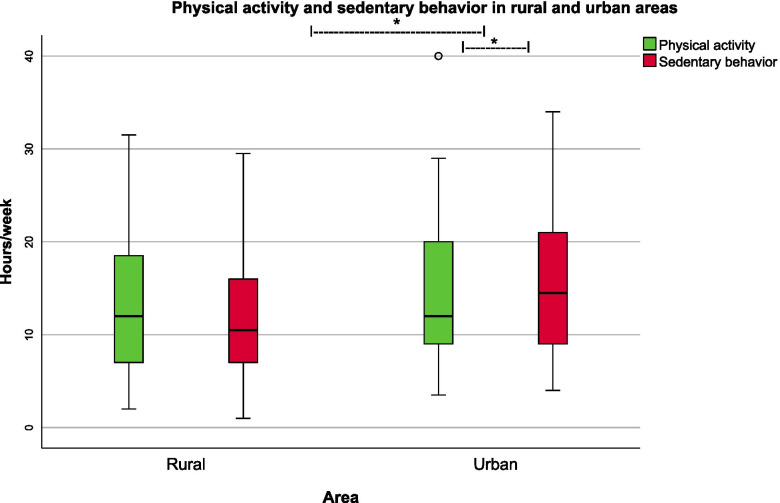
Physical activity and sedentary behaviors. Weekly hours preschoolers spend for physical activity and sedentary behaviors in selected rural and urban regions in Austria, * *p* <0.05 Wilcoxon sign test or Wilcoxon-Mann-Whitney-U test, (survey in Carinthia n = 72, and Vienna n = 58)

In Vienna, hours spent on sedentary behaviors per week are significantly more than physical activity (Wilcoxon sign test, *p*=0.042). In Carinthia, on the other hand, the time for sedentary behaviors and physical activity is comparable (Wilcoxon sign test, *p*=0.128) (Fig. 1).

The median duration of sedentary behaviors is significantly longer in Vienna (14.5 h (4- 34) than in Carinthia (10.75 h (4-30) (*p*=0.01), while there are no differences as for physical activity (*p*=0.746) (Table 1).

**Fig. 2:**
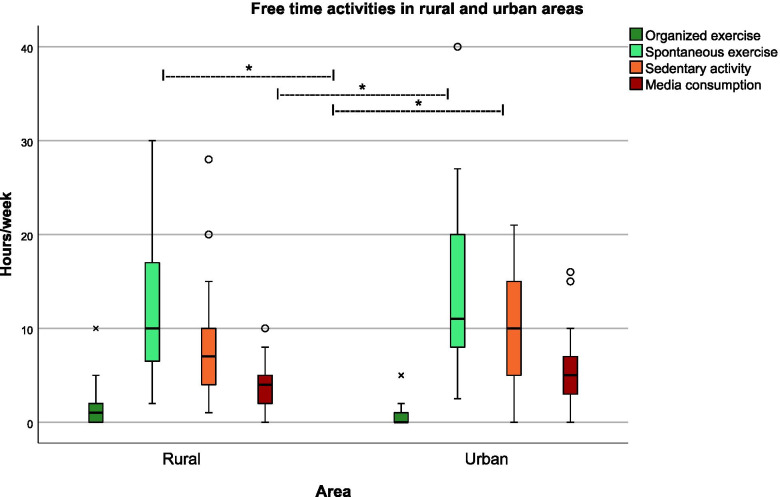
Distribution of free-time activities. Preschoolers’ free-time spent on organized exercise, spontaneous exercise, sedentary activity, and media consumption in weekly hours in selected rural and urban regions of Austria (survey in Carinthia n = 72, and Vienna n = 58), * *p* <0.05 Wilcoxon-Mann-Whitney -U test

Preschoolers who live in the urban area spend more time engaging in sedentary activities (*p*=0.03) and media consumption (*p*=0.007) than rural preschoolers. On the other hand, preschoolers from the rural area practice more organized exercise (*p*=0.001) Fig. 2.

### Free-time activities among the weight classes

To analyze possible associations between physical activity (organized and spontaneous exercise) or sedentary behaviors (sedentary activity and media consumption) and BMI, preschoolers were categorized according to their weight status, i.e. underweight, normal weight, overweight and obese.

Our data show no significant association between BMI and physical activity (*p*=0.187) or BMI and sedentary behaviors (*p*=0.722) (Table 3). An increasing BMI determines a trendwise decrease in the weekly hours of spontaneous exercise (*p*=0.105). There is no association between organized exercise and BMI (*p*=0.308), presumably because of the small number of cases per group (Table 3). Preschoolers of all weight classes spend, on average, twice as much time engaging in sedentary activities than in media consumption (Table 3).

**Table 3:** Weight class and free-time activity in (h/week)

	Underweight	Normalweight	Overweight w/o obesity	Obesity	***p***	***p***-adjust
	(*n* = 6)	(*n* = 108)	(*n* = 9)	(*n* = 5)		
**Physical activity**	19 (7-25)	12 (0-40)	9 (6-21)	7 (5-13)	0.187	0.935
Organized exercise	0 (0-3)	0 (0-10)	1 (0-3)	1 (0-3)	0.308	1
Spontaneous exercise	17.5 (7-25)	10 (2-40)	8 (4-20)	7 (4-10)	0.105	0.63
**Sedentary behaviors**	14.75 (4-18)	13 (1-34)	16.5 (4-30)	8 (6-16)	0.722	1
Sedentary activity	10 (3-15)	7 (0-21)	10 (2-28)	5 (4-15)	0.702	1
Media consumption	3 (1-7)	4 (0-16)	4 (2-10)	3 (1-8)	0.622	1

median (min-max); *p*-value calculated using the Kruskal-Wallis test; *p*-adjust value after Bonferroni correction

### Relationship between BMI and socio-economic factors

As socioeconomic factors might influence the BMI, we addressed this aspect using a linear regression model. After Johnson transformation, linear regression analysis for the dependency of BMI of preschoolers on the explanatory socioeconomic variables such as employment, education level, and age of the father; employment, education level, and age of the mother, as well as age, gender and federal state residency did not show a significant correlation (*p*≤0.05) for any explanatory variables (see Supplementary Table 1).

**Fig. 3:**
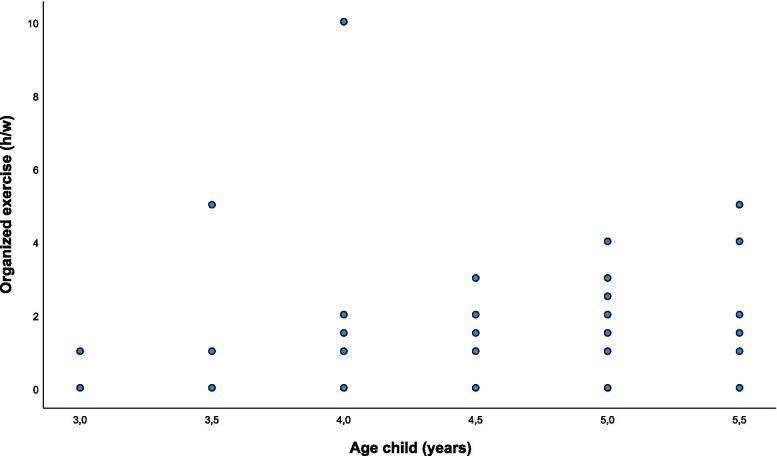
Age dependent time for organized exercise. Correlation of the age of Austrian preschoolers to the amount of free-time spent in organized exercise in a week (survey in Vienna and Carinthia, n = 129)

As a next step we investigated a possible age-specific effect on free-time activities using the Spearman correlation analysis. Age had no significant association with free-time activities per se. A correlation, however, was observed between age and weekly hours dedicated to organized exercise (correlation coefficient Spearman-*Rho* = 0.387), the two being directly proportional (*p* <0.001, Fig. 3).

## Discussion

In this observational cross-sectional study, we investigated the free-time of preschoolers (time outside of nursery hours) from the urban area of Vienna and the rural area of Carinthia.

According to parents’ reports, preschoolers spent approximately as many hours of their free-time doing physical activity as engaging in sedentary behaviors. Media consumption amounts to one-third of the time dedicated to sedentary activities. Preschoolers’ free-time activities in Vienna significantly differ in the fewer hours dedicated to organized exercise and the more extended period reserved for sedentary activity and watching screen-based entertainment. In our study cohort, 7 % of preschoolers in selected areas of Austria were overweight, 3.9 % were obese. No differences regarding the prevalence of overweight and obesity could be found among said regions. No differences were noted in the free-time activities among the weight classes, which might be due to the small sample size. However, organized exercise seems to be trendwise negatively correlated with BMI and might depend on the age of the child. No other socio-economic factors influenced BMI in our cohort.

Obesity rates among preschoolers differ worldwide, with the lowest numbers in developing countries [46] and the highest prevalence among 2- to 5-year-olds (13 %) in the US according to the CDC [47]. A comparison of our data on the prevalence of overweight and obesity with the results of the study by Mayer et al. 2015 [48], focusing on children and adolescents aged 4 to 19, reveals a similar proportion of obesity in children (between 3 % and 5 %). In contrast, our data showed a lower proportion of overweight children, 9 % of boys, 4 % of girls vs. 18 % of boys, 12 % of girls [48]. In our study, the rate of overweight girls was substantially lower. A remark about the discrepancy in our results and the apparent overweight rates decline would be purely speculative but, as parents of obese children might have refused to participate in our study, we consider a selection bias a possible explanation.

As shown in various studies, urban-rural-specific trends of overweight prevalence differ worldwide [32–36]. According to Johnson et al., rural areas of the United States in 2015 were associated with 1.26 higher odds of obesity for 2-19-year-olds compared to urban children [35]. The Austrian participation in the COSI 2017 (8-9 years), however, showed an increased risk in cities [49].

Data on the prevalence of obesity among preschoolers in specific parts of Austria are rare. A Tirolean study revealed 7.6 % of preschoolers to be overweight and 5.5 % to be obese with no differences between mountainous and non-mountainous areas [50]. Despite the results of the Statistics Austria 2014 health survey on adults ranking Carinthia as the region with the second-highest prevalence of overweight and Vienna as the area with the lowest overweight prevalence [51], our study on preschoolers aged 3 to 5 shows no notable contrasts between the two regions being in line with the Tirolean study. This might be explained by the fact that our selected regions are not representative or that being overweight is less common at an early age and rather occurs later in life. This hypothesis is consistent with US data showing obesity to be more common in adolescence (20 % of 12- to 19-year-olds) [47]. Evidence in our study on the degree of urbanization as a risk factor for obesity remains inconclusive and needs to be addressed with a more representative study cohort.

Free-time activities are part of those lifestyle habits that are shaped at a young age and likely maintained later in life, determining a higher or lower BMI development. However, data on the correlation between physical activity and weight in children and adolescents are partially contradictory. According to a review by Prentice-Dunn et al. 2012, sedentary behaviors are positively associated with obesity in most studies [25]. Our findings are in line with other results showing no differences in active leisure habits between obese first-grade children compared to controls [52, 53]. Overweight children might favor sedentary behavior due to poorer motor abilities such as endurance, strength, coordination and balance [54].

Even though the association of physical activity with BMI in preschoolers is evident, further studies are needed. It is equally advisable to encourage an active lifestyle since childhood to prevent unhealthy lifestyle habits, as physical activity often reduces with age [24]. Our results reveal that preschoolers spend an average of 12 h/week (720 min/week, i.e. 102.86 min/day) engaging in physical activity and therefore exceed the 60 min daily minimum suggested by the WHO [55]. However, our findings should be interpreted with caution, as self-report questionnaires might tend to provide overestimations of actual behaviors. According to a systematic review, only 54 % of preschoolers are sufficiently active and meet the recommendations of the WHO [56].

Preschoolers in Austria spend 13 h/week (780 min/week, i.e. 111.43 min/day) engaging in sedentary behaviors. Outside nursery hours, they favor spontaneous exercise and sedentary activities such as painting, handicrafts, and games. According to the parents’ reports their preschool children meet the WHO recommendations of not exceeding 60 min of sedentary screen time per day (median of 4 h per week, 34 min/day) [55]. Therefore, media consumption for children this age amounts to about half of the time allotted for other sedentary activities (median 7 h/w).

With regard to the effects of socioeconomic factors such as residency, recreational activities have significantly different durations in rural and urban areas. In the United States, Euler et al. 2019 observed that teenagers in cities spend less time practicing physical activity and more in sedentary behaviors [34]. We remarked the same pattern in urban areas of Austria where preschoolers engage more in sedentary activities and media consumption, but no significant difference in physical activity were reported. Although our data indicate that preschoolers spend enough time engaging in physical activity in their free-time, the influencing factors such as urban versus rural residency or socio-economic status must be taken into account. Children from disadvantaged homes participate less frequently in free-time physical activity than those from higher-income families [57], and are more likely to do so in adulthood [58]. Thus, the relevance of early life preventive measures and the focus on influencing factors.

### Limitations

Our study is an exploratory analysis and has some limitations. The selection bias, inadequate randomization, and the insufficient sample size from only two of the nine federal states make our results non-representative of the whole country. Accordingly, it is impossible to draw definitive conclusions. Sample size calculation was not performed due to the design of the study and the limited number of nurseries that were willing to participate. One of the limitations might be the questionnaire, which was self-administered, although based on validated study designs. Furthermore, presumably, parents of obese preschoolers did not consent to participate in the study. Also, evaluations on free-time activities are based on the parents’ subjective assessment and could be susceptible to inaccuracy. Due to the small sample size some of the effects might be statistically insignificant. Nonetheless, our efforts lay the foundation for future research to focus on more representative cohorts and provide indispensable insights into this topic.

## Conclusions

Despite the limitations of this study, our results proved that, in urban areas, the duration of sedentary behaviors is significantly longer than physical activity, exposing preschoolers to an unhealthy life-style. Rural preschoolers might follow healthier life style habits as they participate more in organized sports (facilitating weight control) and less sedentary activities. As overweight and obesity affect preschool children, it is compelling to consecrate the utmost attention to obesity prevention at a young age and promote physical activity to tackle obesity and its onset later in life. Future studies could build on our outcomes to address obesity prevention and children’s free-time activities in more representative settings.

## Supplementary Information

**Additional file 1.**

**Additional file 2.**

## Data Availability

The datasets used and analyzed in the current study are available from the corresponding author on reasonable request.

## Abbreviations

BMI: Body mass index
WHO: World health organization

## References

[1] WHO. Obesity and overweight. World Health Organization. http://www.who.int/news-room/fact-sheets/detail/obesity-and-overweight. Accessed 3 Sep 2018.

[2] Kiefer I, Rieder A, Rathmanner T, Meidlinger B, Baritsch C, Lawrence K, et al. Erster Österreichischer Adipositasbericht 2006. Grundlagen für zukünftige Handlungsfelder: Kinder, Jugendliche, Erwachsene. Verein Altern mit Zukunft, Herausgeber; 2016.

[3] WHO - Body mass index - BMI. WHO Regional Office for Europe. http://www.euro.who.int/en/health-topics/disease-prevention/nutrition/a-healthy-lifestyle/body-mass-index-bmi. Accessed 4 Sep 2018.

[4] Kromeyer-Hauschild K, Wabitsch M, Kunze D (2001). Perzentile für den Body-mass-Index für das Kindes-und Jugendalter unter Heranziehung verschiedener deutscher Stichproben. Monatsschr Kinderheilkd.

[5] Cole TJ, Bellizzi MC, Flegal KM, Dietz WH. Establishing a standard definition for child overweight and obesity worldwide: international survey. BMJ. 320:1240–3. 10.1136/bmj.320.7244.1240 PMC27365 10797032

[6] Definition der Adipositas (AGA). Arbeitsgemeinschaft Adipositas im Kindes- und Jugendalter. http://www.aga.adipositas-gesellschaft.de/index.php?id=8. Accessed 5 Sep 2018.

[7] Wabitsch M, Kunze D (federführend für die AGA). Konsensbasierte (S2) Leitlinie zur Diagnostik, Therapie und Prävention von Übergewicht und Adipositas im Kindes- und Jugendalter. 2015. www.a-g-a.de. Accessed 31 Aug 2020.

[8] Seese B. Pathophysiologie der Adipositas. CME-Verlag; 2018. https://www.cme-kurs.de/cdn2/pdf/Handout_Adipositas.pdf. Accessed 16 Apr 2021.

[9] Liao X-P, Yu Y, Marc I, Dubois L, Abdelouahab N, Bouchard L (2019). Prenatal determinants of childhood obesity: a review of risk factors. Can J Physiol Pharmacol.

[10] Page KA, Luo S, Wang X, Chow T, Alves J, Buchanan TA (2019). Children Exposed to Maternal Obesity or Gestational Diabetes Mellitus During Early Fetal Development Have Hypothalamic Alterations That Predict Future Weight Gain. Dia Care.

[11] Mueller NT, Whyatt R, Hoepner L, Oberfield S, Dominguez-Bello MG, Widen EM (2015). Prenatal exposure to antibiotics, cesarean section and risk of childhood obesity. Int J Obes.

[12] Morgen CS, Ängquist L, Baker JL, Andersen AMN, Michaelsen KF, Sørensen TIA (2018). Prenatal risk factors influencing childhood BMI and overweight independent of birth weight and infancy BMI: a path analysis within the Danish National Birth Cohort. Int J Obes.

[13] WHO. Obesity. http://www.who.int/topics/obesity/en/. Accessed 3 Mar 2017.

[14] Kelsey MM, Zaepfel A, Bjornstad P, Nadeau KJ (2014). Age-Related Consequences of Childhood Obesity. Gerontology.

[15] Müller R (2013). Psychische Folgeprobleme der Adipositas. Therapeutische Umschau.

[16] Cramer P, Steinwert T (1998). Thin is good, fat is bad: How early does it begin?. J Appl Dev Psychol.

[17] Harriger JA, Thompson JK (2012). Psychological consequences of obesity: Weight bias and body image in overweight and obese youth. Int Review Psychiatry.

[18] Singh AS, Mulder C, Twisk JWR, Van Mechelen W, Chinapaw MJM (2008). Tracking of childhood overweight into adulthood: a systematic review of the literature. Obes Rev.

[19] Döring N, Mayer S, Rasmussen F, Sonntag D (2016). Economic Evaluation of Obesity Prevention in Early Childhood: Methods, Limitations and Recommendations. IJERPH.

[20] Gao Z, Chen S, Huang CC, Stodden DF, Xiang P (2017). Investigating elementary school children’s daily physical activity and sedentary behaviours during weekdays. J Sports Sci.

[21] Arundell L, Hinkley T, Veitch J, Salmon J (2015). Contribution of the After-School Period to Children’s Daily Participation in Physical Activity and Sedentary Behaviours. PLoS ONE.

[22] Herman KM, Craig CL, Gauvin L, Katzmarzyk PT (2009). Tracking of obesity and physical activity from childhood to adulthood: The Physical Activity Longitudinal Study. International Journal of Pediatric Obesity.

[23] Dhar P, Robinson C (2016). Physical activity and childhood obesity. Appl Econ Lett.

[24] Biddle SJ, Gorely T, Stensel DJ (2004). Health-enhancing physical activity and sedentary behaviour in children and adolescents. J Sports Sci.

[25] Prentice-Dunn H, Prentice-Dunn S (2012). Physical activity, sedentary behavior, and childhood obesity: A review of cross-sectional studies. Psychology Health Medicine.

[26] Hu FB (2003). Television Watching and Other Sedentary Behaviors in Relation to Risk of Obesity and Type 2 Diabetes Mellitus in Women. JAMA.

[27] Tandon PS, Zhou C, Sallis JF, Cain KL, Frank LD, Saelens BE (2012). Home environment relationships with children’s physical activity, sedentary time, and screen time by socioeconomic status. Int J Behav Nutr Phys Act.

[28] Wilkie HJ, Standage M, Gillison FB, Cumming SP, Katzmarzyk PT (2018). The home electronic media environment and parental safety concerns: relationships with outdoor time after school and over the weekend among 9–11 year old children. BMC Public Health.

[29] Pearson N, Braithwaite RE, Biddle SJH, van Sluijs EMF, Atkin AJ (2014). Associations between sedentary behaviour and physical activity in children and adolescents: a meta-analysis: Active and sedentary behaviours in youth. Obes Rev.

[30] Biddle SJ, Gorely T, Marshall SJ, Murdey I, Cameron N (2004). Physical activity and sedentary behaviours in youth: issues and controversies. J Royal Soc Promotion Health.

[31] Zhang Y-X, Wang Z-X, Zhao J-S, Chu Z-H (2016). Prevalence of Overweight and Obesity among Children and Adolescents in Shandong, China: Urban–Rural Disparity. J Trop Pediatr.

[32] Trivedi T, Liu J, Probst J, Merchant A, Jhones S, Martin AB (2015). Obesity and obesity-related behaviors among rural and urban adults in the USA. Rural Remote Health.

[33] McCormack LA, Meendering J (2016). Diet and Physical Activity in Rural vs Urban Children and Adolescents in the United States: A Narrative Review. Journal of the Academy of Nutrition Dietetics.

[34] Euler R, Jimenez EY, Sanders S, Kuhlemeier A, Van Horn ML, Cohen D (2019). Rural–Urban Differences in Baseline Dietary Intake and Physical Activity Levels of Adolescents. Prev Chronic Dis.

[35] Johnson JA, Johnson AM (2015). Urban-Rural Differences in Childhood and Adolescent Obesity in the United States: A Systematic Review and Meta-Analysis. Childhood Obesity.

[36] Cohen SA, Cook SK, Kelley L, Foutz JD, Sando TA (2017). A Closer Look at Rural-Urban Health Disparities: Associations Between Obesity and Rurality Vary by Geospatial and Sociodemographic Factors: Rural-Urban Disparities: Moderation by Place & SES. J Rural Health.

[37] Statistik Austria. Urban-Rural-Typologie. https://www.statistik.at/web_de/klassifikationen/regionale_gliederungen/stadt_land/index.html. Accessed 28 Aug 2020.

[38] Food and Agriculture Organization of the United Nations. Guidelines on defining rural areas and compiling indicators for development policy. 2018. http://www.fao.org/3/ca6392en/ca6392en.pdf. Accessed 28 Aug 2020.

[39] Neuhauser H, Schienkiewitz A, Schaffrath Rosario A, Dortschy R, Kurth B-M. Referenzperzentile für anthropometrische Maßzahlen und Blutdruck aus der Studie zur Gesundheit von Kindern und Jugendlichen in Deutschland (KiGGS). Robert Koch-Institut. 2013:129.https://www.rki.de/DE/Content/Gesundheitsmonitoring/Gesundheitsberichterstattung/GBEDownloadsB/KiGGS_Referenzperzentile.pdf?_blob=publicationFile. Accessed 16 Apr 2021.

[40] Lynch E, Liu K, Spring B, Hankinson A, Wei GS, Greenland P (2007). Association of Ethnicity and Socioeconomic Status with Judgments of Body Size: The Coronary Artery Risk Development in Young Adults (CARDIA) Study. Am J Epidemiol.

[41] Krieger N, Williams DR, Moss NE (1997). Measuring Social Class in US Public Health Research: Concepts, Methodologies, and Guidelines. Annu Rev Public Health.

[42] Auhuber L, Vogel M, Grafe N, Kiess W, Poulain T (2019). Leisure Activities of Healthy Children and Adolescents. IJERPH.

[43] Strand BH, Kunst A (2006). Childhood Socioeconomic Position and Cause-specific Mortality in Early Adulthood. Am J Epidemiol.

[44] Hemmerich W, StatistikGuru: Johnson Transformation berechnen. 2016. https://statistikguru.de/rechner/johnson-transformation-berechnen.html.

[45] Elm E, Altmann DG, Egger M, Pocock SC, Gøtzsche PC (2008). Das Strengthening the Reporting of Observational Studies in Epidemiology (STROBE-) Statement: Leitlinien für das Berichten von Beobachtungsstudien. Internist.

[46] Gupta N, Goel K, Shah P, Misra A (2012). Childhood Obesity in Developing Countries: Epidemiology, Determinants, and Prevention. Endocr Rev.

[47] Craig M, Hales MD, Margaret D, Carroll MSPH, Cheryl D, Fryar MSPH, Ogden CL, Ph.D. Prevalence of Obesity Among Adults and Youth: United States, 2015–2016. NCHS Data Brief. 2017. https://www.cdc.gov/obesity/data/childhood.html. Accessed 16 Apr 2021. 29155689

[48] Mayer M, Gleiss A, Häusler G, Borkenstein M, Kapelari K, Köstl G (2015). Weight and body mass index (BMI): current data for Austrian boys and girls aged 4 to under 19 years. Ann Hum Biol.

[49] Bundesministerium für Gesundheit und Frauen. Childhood Obesity Surveillance Initiative (COSI). 2017. https://broschuerenservice.sozialministerium.at/Home/Download?publicationId=521. Accessed 16 Apr 2021.

[50] Greier K, Riechelmann H, Burtscher M (2014). Prevalence of obesity and motor performance capabilities in Tyrolean preschool children. Wien Klin Wochenschr.

[51] Statistik Austria. Bevölkerung in Privathaushalten im Alter von 15 und mehr Jahren. BMI Body Mass Index. 2015. https://www.statistik.at/web_de/statistiken/menschen_und_gesellschaft/gesundheit/gesundheitsdeterminanten/bmi_body_mass_index/025420.html. Accessed 13 Sep 2018.

[52] Tomaz SA, Prioreschi A, Watson ED, McVeigh JA, Rae DE, Jones RA (2019). Body Mass Index, Physical Activity, Sedentary Behavior, Sleep, and Gross Motor Skill Proficiency in Preschool Children From a Low- to Middle-Income Urban Setting. J Phys Act Health.

[53] Vale SM, Santos RM, da Soares-Miranda C, Moreira LM, Ruiz CM, Mota JR (2010). Objectively measured physical activity and body mass index in preschool children. Int J Pediatr.

[54] Graf C, Koch B, Dordel S, Schindler-Marlow S, Icks A, Schüller A (2004). Physical activity, leisure habits and obesity in first-grade children. European Journal of Cardiovascular Prevention Rehabilitation.

[55] World Health Organization. Guidelines on physical activity, sedentary behaviour and sleep for children under 5 years of age. 2019. https://apps.who.int/iris/handle/10665/311664. Accessed 16 Apr 2021. 31091057

[56] Tucker P (2008). The physical activity levels of preschool-aged children: A systematic review. Early Childhood Research Quarterly.

[57] Stalsberg R, Pedersen AV (2010). Effects of socioeconomic status on the physical activity in adolescents: a systematic review of the evidence: Effects of socioeconomic status on the physical activity in adolescents. Scandinavian Journal of Medicine Science in Sports.

[58] Elhakeem A, Cooper R, Bann D, Hardy R (2015). Childhood socioeconomic position and adult leisure-time physical activity: a systematic review. Int J Behav Nutr Phys Act.

