# Supplementary material for: A cross-sectional, exploratory survey on health-relevant free-time activities and body mass index in preschool children in urban and rural settings of Austria
Source: BMC Pediatr. 2021 Nov 6;21:495. doi: 10.1186/s12887-021-02972-x (PMC8571826; doi:10.1186/s12887-021-02972-x)
Supplement: Supplementary file 1 — Additional file 1. [file 12887_2021_2972_MOESM1_ESM.docx]

**Supplementary Data and figures**


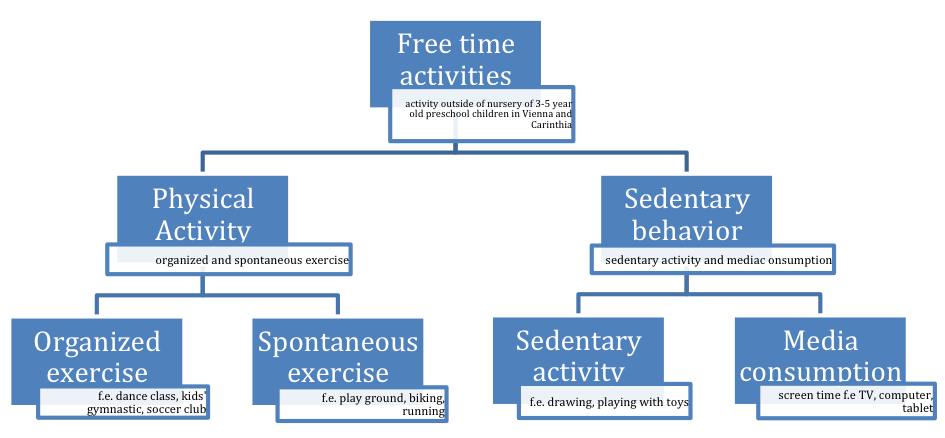
Supplementary Figure 1: Clarification of terms and overview of the parameters, including questionnaires on 3-5-year-old preschoolers, collected from selected rural and urban regions of Austria, with explanatory examples (Vienna and Carinthia)

| Supplementary Table 1. Regression coefficient B and significance of the linear  regression analysis of the BMI dependent variable after Johnson transformation | | | | |
| --- | --- | --- | --- | --- |
|  | *B* | 95% confidence  interval | Beta | *p*-value |
| Area | -0.134 | -0.505, 0.238 | -0-063 | 0.477 |
| Age mother | -0.053 | -0.231, 0.125 | -0.052 | 0.557 |
| Education mother | 0.146 | -0.081, 0.374 | 0.113 | 0.206 |
| Employment mother | 0.097 | -0.391, 0.584 | 0.035 | 0.695 |
| Age father | -0.094 | -0.278, 0.098 | -0.084 | 0.347 |
| Education father | -0.094 | -0.317, 0.129 | -0.076 | 0.405 |
| Employment father | 0.262 | -0.550, 1.075 | 0.058 | 0.524 |
| Age child | 0.101 | -0.083, 0.284 | 0.096 | 0.280 |
| Gender | -0.094 | -0.459,0.279 | -0.043 | 0.630 |
