# Supplementary material for: A cross-sectional, exploratory survey on health-relevant free-time activities and body mass index in preschool children in urban and rural settings of Austria
Source: BMC Pediatr. 2021 Nov 6;21:495. doi: 10.1186/s12887-021-02972-x (PMC8571826; doi:10.1186/s12887-021-02972-x)
Supplement: Supplementary file 2 — Additional file 2. [file 12887_2021_2972_MOESM2_ESM.docx]

**Supplementary data - Questionnaire**

**Mother** Age: _________ years

Highest education level:

A: < High school diploma B: High school diploma C: University

Employment?

Yes No

**Father** Age: _________ years

Highest education level:

A: < High school diploma B: High school diploma C: University

Employment?

Yes No

**Child**

Age: a: 3 years b: 3.5 years

c: 4 years d: 4.5 years

e: 5 years f: 5.5 years

Sex: a: male b: female

Chronic disease? Yes No

Preterm birth? Yes No

Birth order?

Firstborn Lastborn Other: ______________

**How much time does the child spend on average per week for:**

**Organized exercise** (such as consistent attendance, i.e., dance class, or gymnastics)?

________________ hours per week

**Spontaneous exercise** (additional physical exertion like running, biking, playground activities)?

_______________ hours per week

**Sedentary activity** (such as playing with toys, drawing)?

______________ hours per week

**Media consumption**?

______________ hours per week
